# Supplementary material for: Derivation of Genetically Defined Murine Hepatoblastoma Cell Lines with Angiogenic Potential
Source: Cancers (Basel). 2025 Sep 14;17(18):3002. doi: 10.3390/cancers17183002 (PMC12468702; doi:10.3390/cancers17183002)
Supplement: Supplementary file 1 [file cancers-17-03002-s001.zip › cancers-3808479-Supplementary file 6.pptx]

## Slide 1
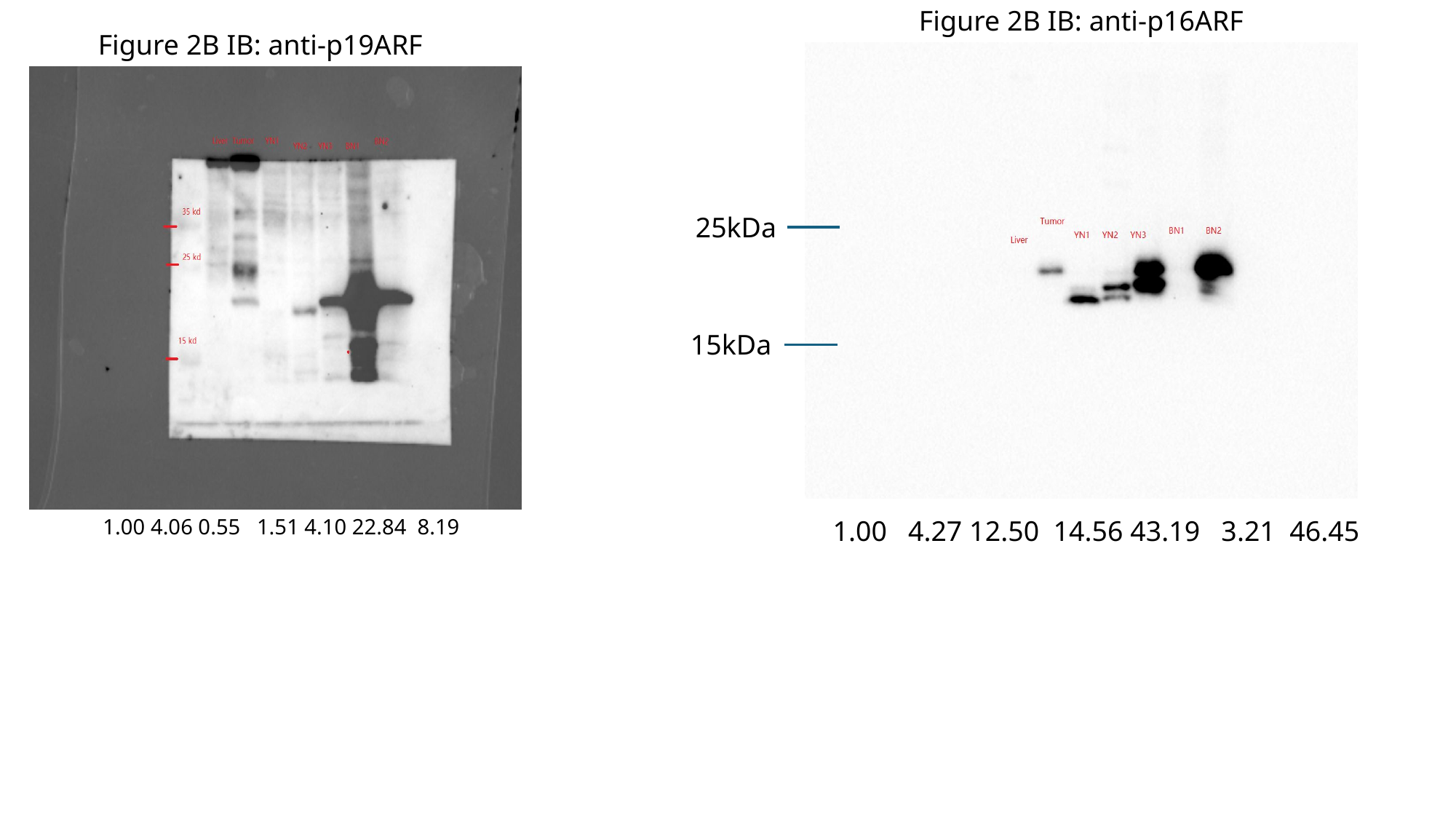

Figure 2B IB: anti-p16ARF
Figure 2B IB: anti-p19ARF
25kDa
15kDa
1.00 4.06 0.55 1.51 4.10 22.84 8.19
1.00 4.27 12.50 14.56 43.19 3.21 46.45

## Slide 2
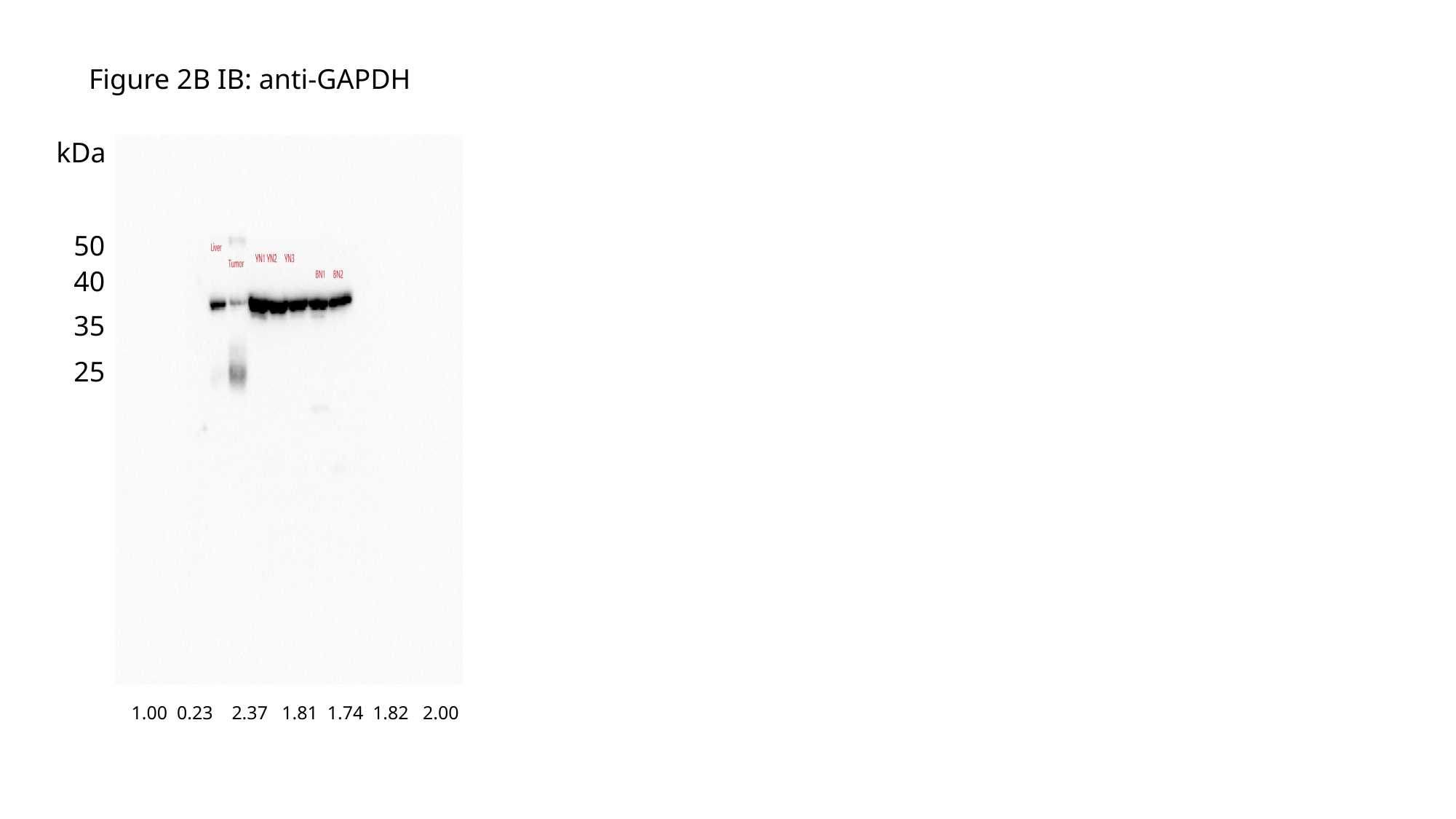

Figure 2B IB: anti-GAPDH
kDa
50
40
35
25
1.00 0.23 2.37 1.81 1.74 1.82 2.00

## Slide 3
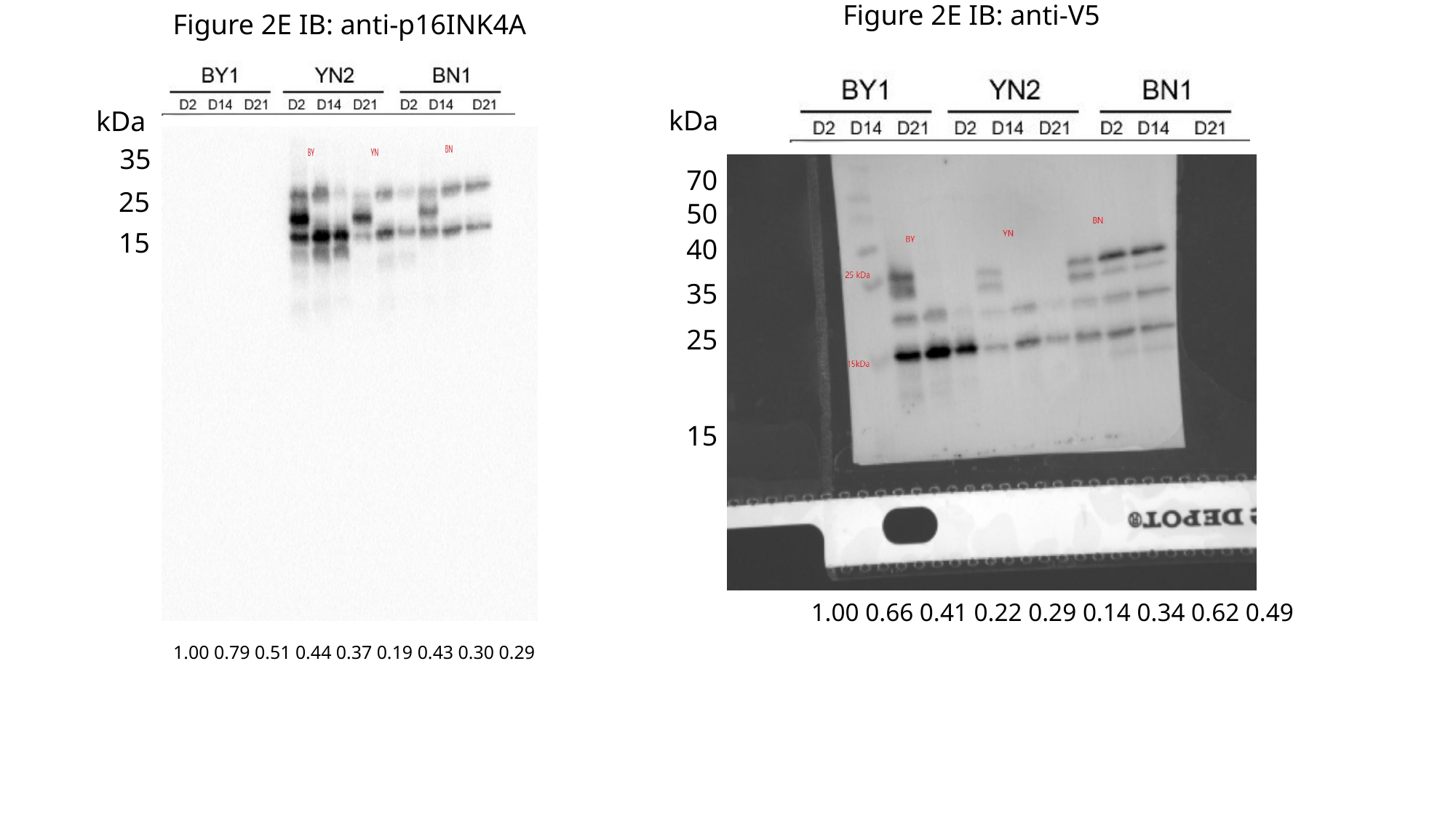

Figure 2E IB: anti-V5
Figure 2E IB: anti-p16INK4A
kDa
70
50
40
35
25
15
kDa
35
25
15
1.00 0.66 0.41 0.22 0.29 0.14 0.34 0.62 0.49
1.00 0.79 0.51 0.44 0.37 0.19 0.43 0.30 0.29

## Slide 4
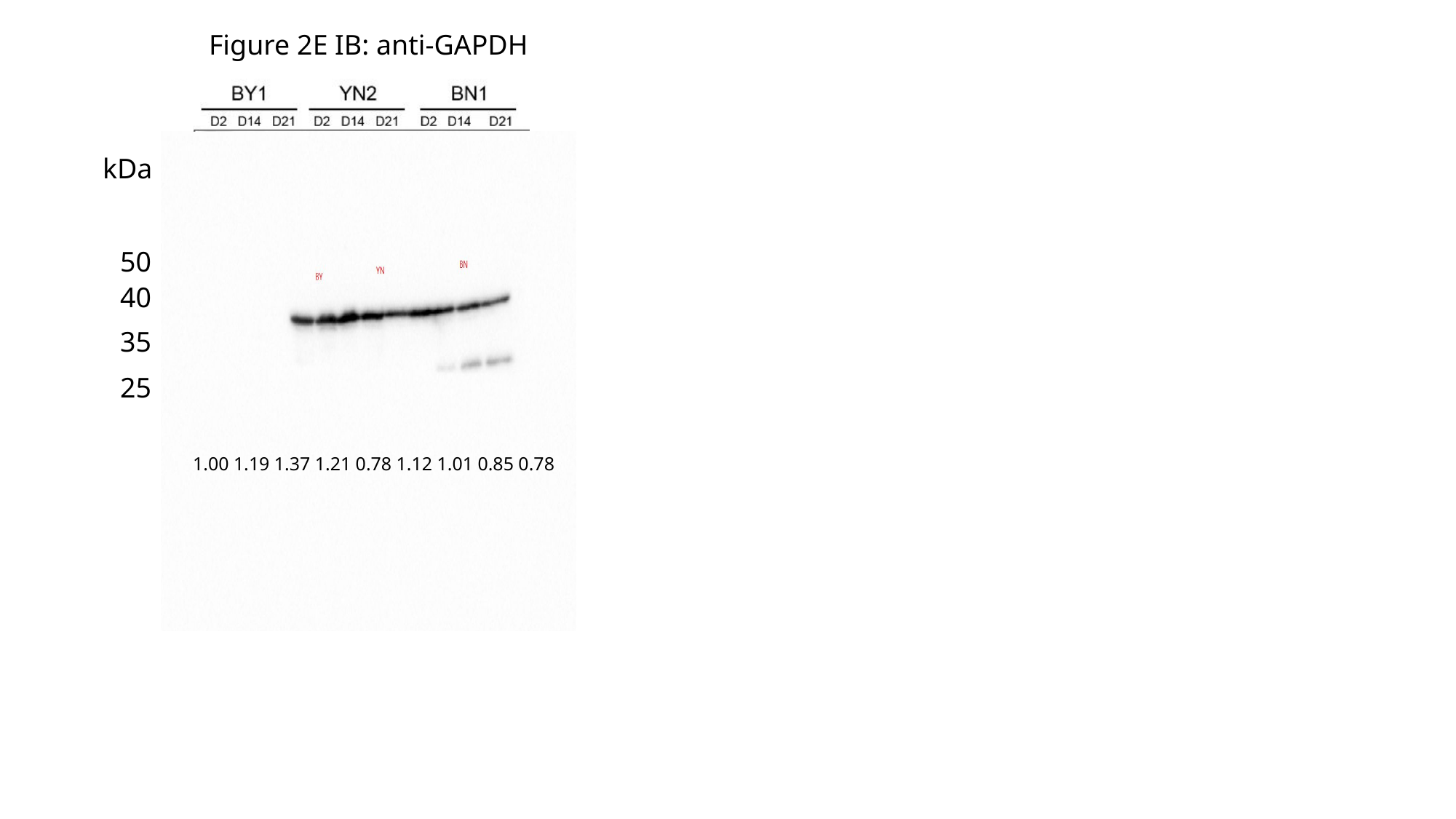

Figure 2E IB: anti-GAPDH
kDa
50
40
35
25
1.00 1.19 1.37 1.21 0.78 1.12 1.01 0.85 0.78

## Slide 5
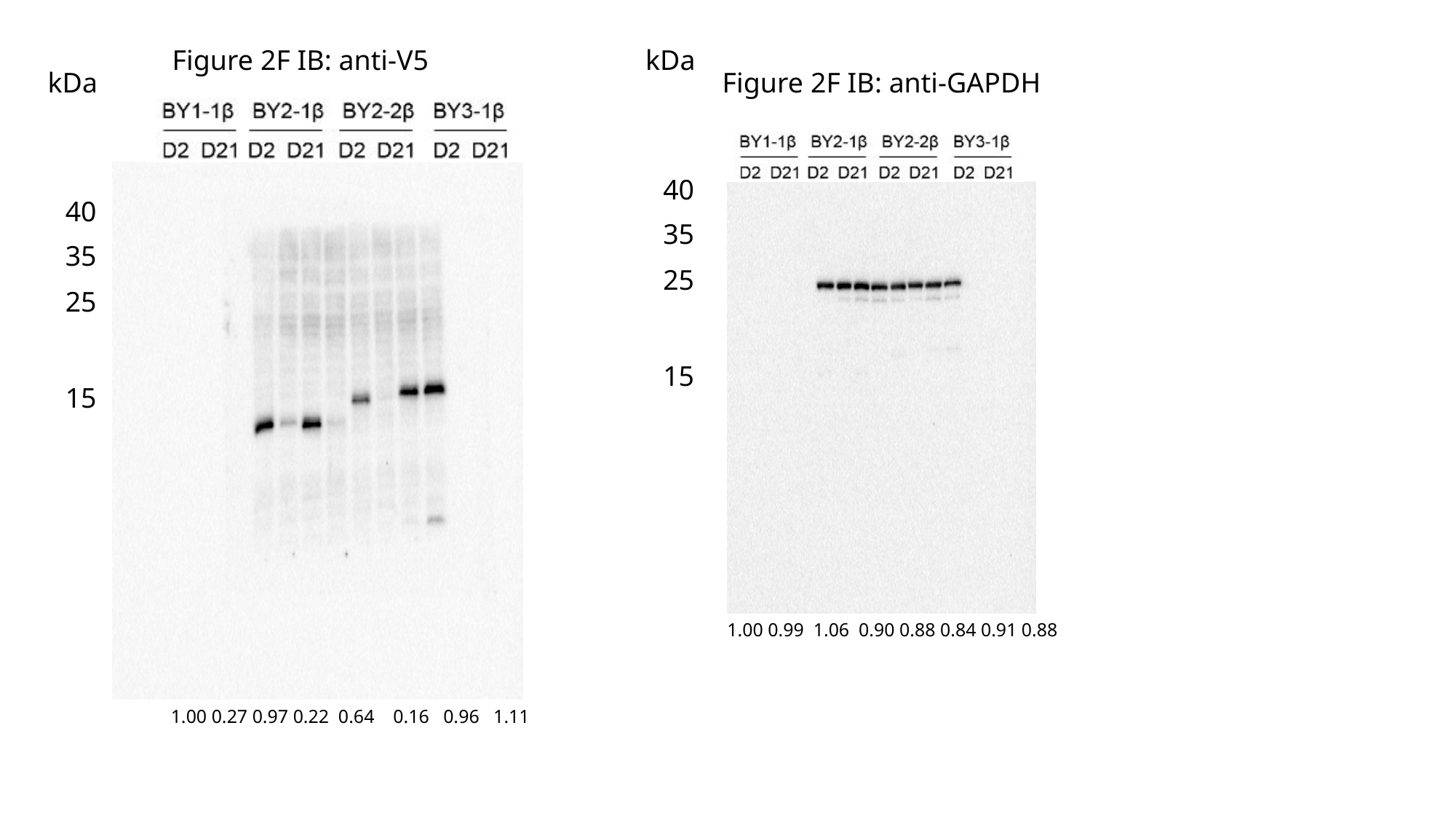

Figure 2F IB: anti-V5
kDa
40
35
25
15
kDa
40
35
25
15
Figure 2F IB: anti-GAPDH
1.00 0.99 1.06 0.90 0.88 0.84 0.91 0.88
1.00 0.27 0.97 0.22 0.64 0.16 0.96 1.11

## Slide 6
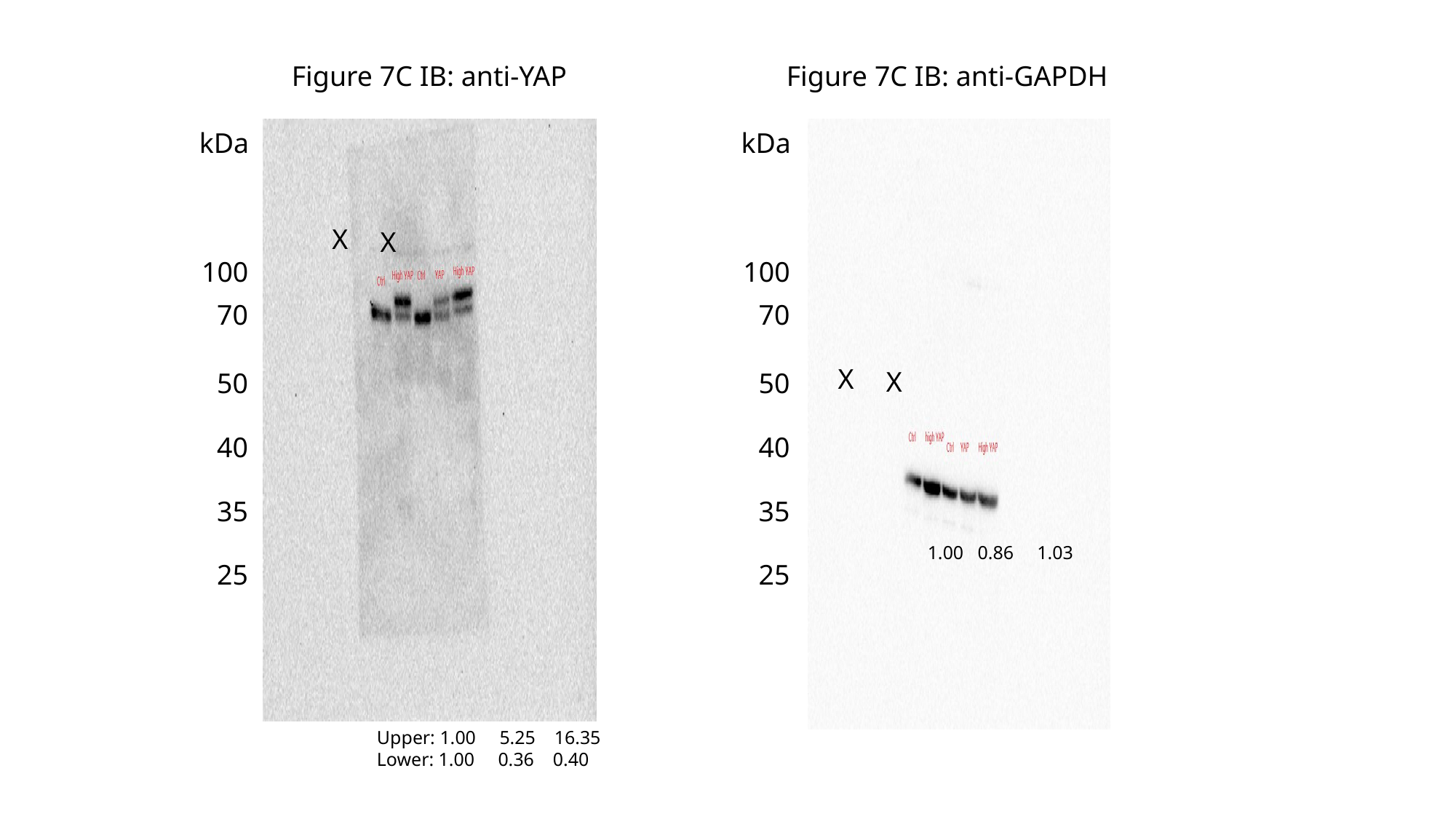

Figure 7C IB: anti-YAP
Figure 7C IB: anti-GAPDH
kDa
100
70
50
40
35
25
kDa
100
70
50
40
35
25
X
X
X
X
1.00 0.86 1.03
Upper: 1.00 5.25 16.35
Lower: 1.00 0.36 0.40
