# Supplementary material for: Derivation of Genetically Defined Murine Hepatoblastoma Cell Lines with Angiogenic Potential
Source: Cancers (Basel). 2025 Sep 14;17(18):3002. doi: 10.3390/cancers17183002 (PMC12468702; doi:10.3390/cancers17183002)
Supplement: Supplementary file 1 [file cancers-17-03002-s001.zip › cancers-3808479_Supplementary_legend.docx]

**Supplementary Materials:**

**Supplementary Figure S1**: Additional examples of H&E-stained sections of subcutaneous tumors generated by the indicated cell lines.

**Supplementary Figure S2**: Additional examples of H&E-stained sections of tumors generated in lungs by the indicated cell lines following tail vein injection.

**Supplementary File 1**: The most commonly identified Cdkn2a exon 2 mutations and their frequencies in immortalized BY and BN cell lines.

**Supplementary File 2**: List of 1,853 EC-specific, non-redundant murine genes (statistical output from DESeq2 analysis; corresponds to Figures 5B, 6G and 6H).

**Supplementary File 3**: List of the unique subset of EC-specific genes expressed in normal murine livers (statistical output from DESeq2 analysis; corresponds to Figure 5B Liver EC-specific gene).

**Supplementary File 4**: List of the 178 hypoxia-responsive genes (statistical output from DESeq2 analysis; corresponds to Figure 6C).

**Supplementary File 5**: List of the 194 liver-specific genes (statistical output from DESeq2 analysis; corresponds to Figure 6E).

**Supplementary File 6:** Original Western Blot images.
