# Supplementary figures and images for: Derivation of Genetically Defined Murine Hepatoblastoma Cell Lines with Angiogenic Potential
Source: Cancers (Basel). 2025 Sep 14;17(18):3002. doi: 10.3390/cancers17183002 (PMC12468702; doi:10.3390/cancers17183002)

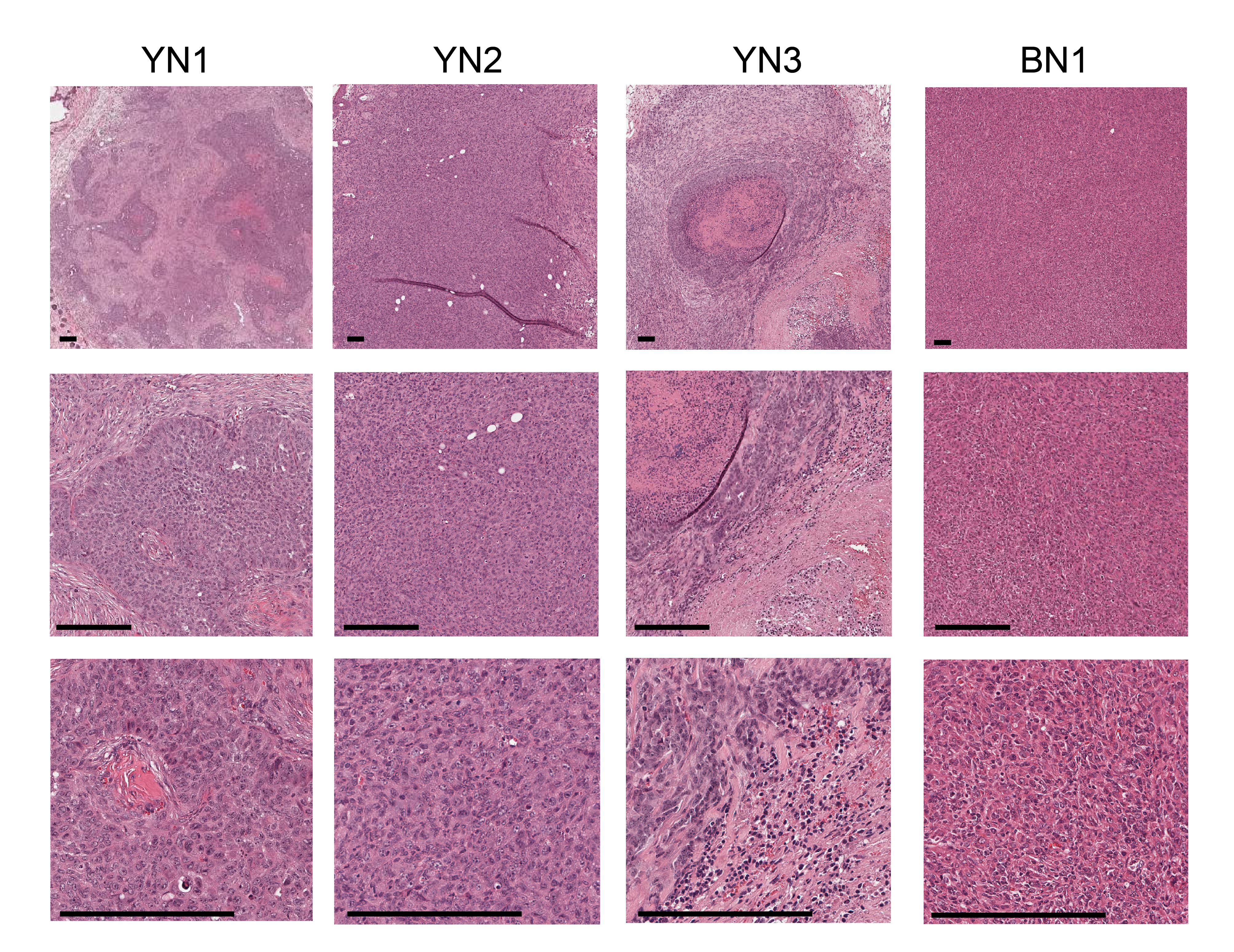

Supplement: Supplementary file 1 [file cancers-17-03002-s001.zip › cancers-3808479-Supplementary Figure S1.jpg]

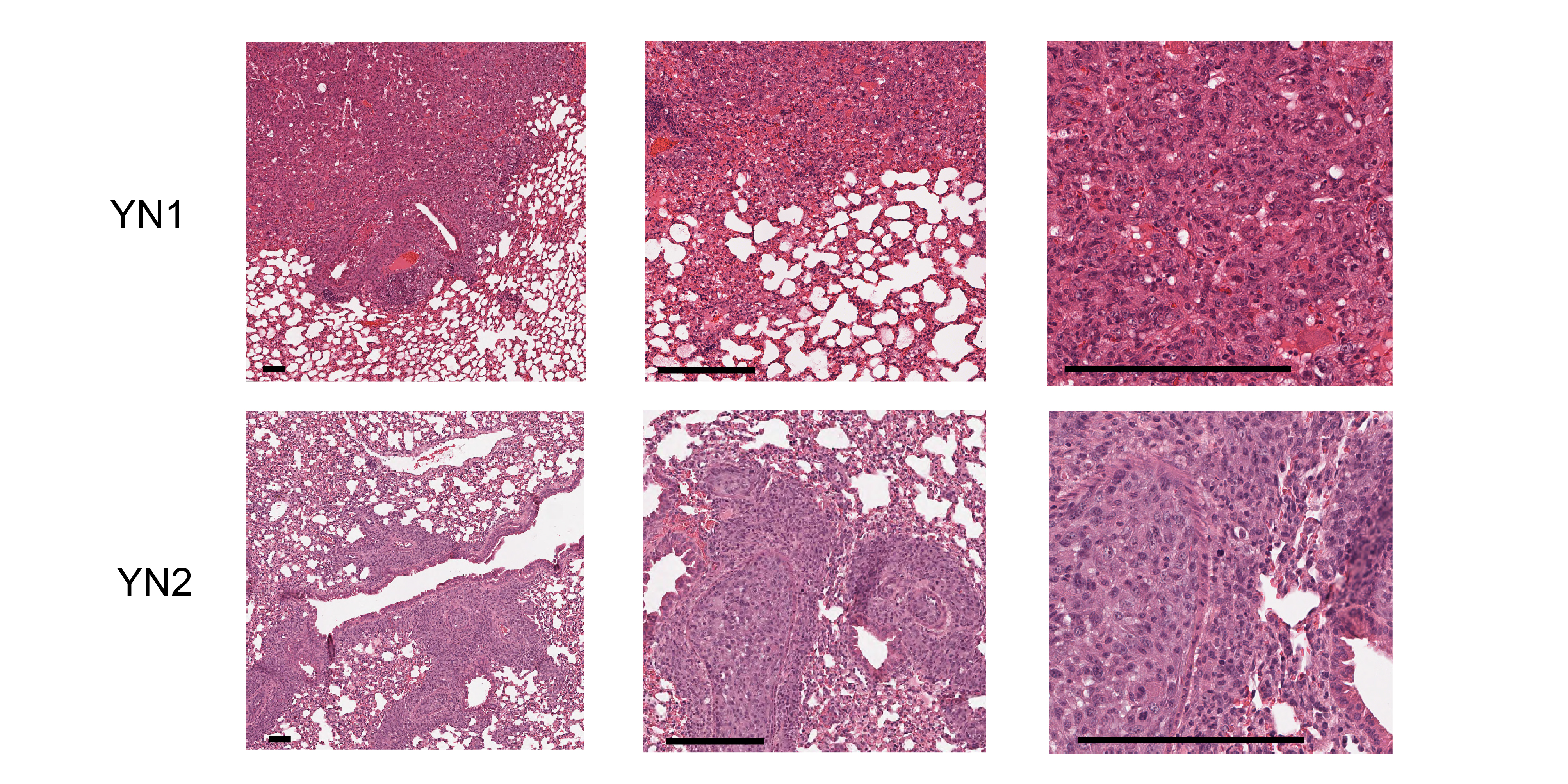

Supplement: Supplementary file 1 [file cancers-17-03002-s001.zip › cancers-3808479-Supplementary Figure S2.jpg]
